# (Picolinato-κ^2^ *N*,*O*)[tris(2-isopropyl-1*H*-imidazol-4-yl-κ*N* ^3^)phosphane]cobalt(II) nitrate

**DOI:** 10.1107/S1600536812004485

**Published:** 2012-02-10

**Authors:** Guido J. Reiss, Markus Börgardts, Peter C. Kunz

**Affiliations:** a Institut für Anorganische Chemie und Strukturchemie, Lehrstuhl II: Material- und Strukturforschung, Heinrich-Heine-Universität Düsseldorf, Universitätsstrasse 1, D-40225 Düsseldorf, Germany; b Institut für Anorganische Chemie und Struktur­chemie, Lehrstuhl I: Bioanorganische Chemie, Heinrich-Heine-Universität Düsseldorf, Universitätsstrasse 1, D-40225 Düsseldorf, Germany; c Institut für Pharmazeutische und Medizinische Chemie, Heinrich-Heine-Universität Düsseldorf, Universitätsstrasse 1, D-40225 Düsseldorf, Germany

## Abstract

Single crystals of the title compound, [Co(C_6_H_4_NO_2_)(C_18_H_27_N_6_P)]NO_3_, were obtained from the reaction of nitrato[tris­(2-isopropyl­imidazol-4-yl)phosphane]cobalt(II) nitrate with picolinic acid in the presence of potassium *tert*-butoxide as base. The coordination polyhedron around the central Co^II^ ion is about halfway between square-pyramidal and trigonal-bipyramidal geometry. In the structure, the nitrate counter-anion is connected by N—H⋯O hydrogen bonding to the complex cation. Additionally, the complex cations form one-dimensional chains along [010] by hydrogen bonding of the NH group of an imidazole ring to the picolinate group of a neighbouring complex cation.

## Related literature

For the synthesis of the title compound, see: Kunz *et al.* (2011 ▶). For structures of related complexes, see: Tekeste & Vahrenkamp (2006 ▶); Kunz *et al.* (2011 ▶). For background to this class of compound, see: Kunz *et al.* (2003 ▶, 2007 ▶, 2008 ▶, 2009 ▶, 2011 ▶); Kunz & Kläui (2007 ▶). For geometric parameters of hydrogen bonding, see: Steiner (2002 ▶). 
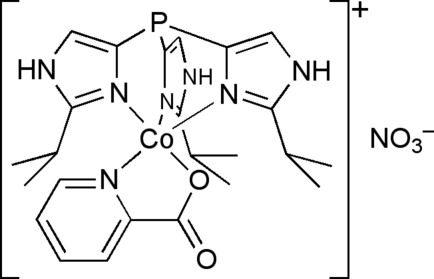


## Experimental

### 

#### Crystal data

[Co(C_6_H_4_NO_2_)(C_18_H_27_N_6_P)]NO_3_
*M* *_r_* = 601.47
Monoclinic, 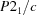

*a* = 15.4012 (5) Å
*b* = 10.7035 (3) Å
*c* = 17.8548 (5) Å
β = 90.491 (3)°
*V* = 2943.21 (15) Å^3^
*Z* = 4
Mo *K*α radiation
μ = 0.68 mm^−1^
*T* = 292 K
0.60 × 0.58 × 0.30 mm

#### Data collection

Oxford Diffraction Xcalibur Eos diffractometer
Absorption correction: gaussian (*CrysAlis PRO*; Oxford Diffraction, 2009 ▶) *T*_min_ = 0.67, *T*_max_ = 0.82
12068 measured reflections
5760 independent reflections
4526 reflections with *I* > 2σ(*I*)
*R*_int_ = 0.022

#### Refinement

*R*[*F* ^2^ > 2σ(*F* ^2^)] = 0.042
*wR*(*F* ^2^) = 0.086
*S* = 1.01
5760 reflections
370 parameters
H atoms treated by a mixture of independent and constrained refinement
Δρ_max_ = 0.43 e Å^−3^
Δρ_min_ = −0.30 e Å^−3^

### 

Data collection: *CrysAlis PRO* (Oxford Diffraction, 2009 ▶); cell refinement: *CrysAlis PRO*; data reduction: *CrysAlis PRO*; program(s) used to solve structure: *SHELXS97* (Sheldrick, 2008 ▶); program(s) used to refine structure: *SHELXL97* (Sheldrick, 2008 ▶); molecular graphics: *DIAMOND* (Brandenburg, 2010 ▶); software used to prepare material for publication: *publCIF* (Westrip, 2010 ▶).

## Supplementary Material

Crystal structure: contains datablock(s) I, global. DOI: 10.1107/S1600536812004485/nc2266sup1.cif

Structure factors: contains datablock(s) I. DOI: 10.1107/S1600536812004485/nc2266Isup2.hkl

Supplementary material file. DOI: 10.1107/S1600536812004485/nc2266Isup3.mol

Additional supplementary materials:  crystallographic information; 3D view; checkCIF report

## Figures and Tables

**Table:** 

Co1—O1	1.9885 (17)
Co1—N5	2.060 (2)
Co1—N3	2.070 (2)
Co1—N1	2.094 (2)
Co1—N7	2.154 (2)

**Table:** 

O1—Co1—N5	118.91 (8)
O1—Co1—N3	139.21 (8)
N5—Co1—N3	100.43 (8)
O1—Co1—N1	101.22 (7)
N5—Co1—N1	90.61 (8)
N3—Co1—N1	87.87 (8)
O1—Co1—N7	78.07 (7)
N5—Co1—N7	92.77 (8)
N3—Co1—N7	90.45 (8)
N1—Co1—N7	176.45 (8)

**Table 2:** Hydrogen-bond geometry (Å, °)

*D*—H⋯*A*	*D*—H	H⋯*A*	*D*⋯*A*	*D*—H⋯*A*
N2—H02⋯O2^i^	0.78 (3)	2.01 (3)	2.786 (3)	174 (3)
N4—H04⋯O3	0.82 (3)	1.98 (3)	2.791 (3)	169 (3)

Symmetry code: (i) 
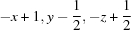
.

**Fig. 1.**
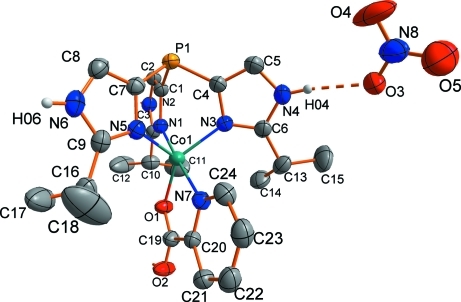
The structure of the asymmetric unit, showing 40% probability displacement ellipsoids. Hydrogen bonding is shown as dashed lines. Hydrogen atoms are shown as spheres of arbitrary radius. Non-acidic hydrogen atoms are omitted for clarity.

**Fig. 2.**
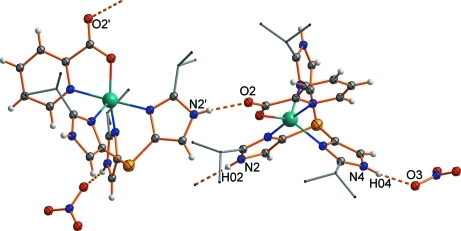
Part of the hydrogen bonded chain (hydrogen bonding is shown as dashed lines, for clarity the isopropy groups are drawn as wires without hydrogen atoms; ' = 1 - x, 0.5 + y, 0.5 - z).

## supplementary crystallographic information

### Comment

Zinc complexes of tripodal *N,N,N* ligands are interesting model compounds for the active sites found in many zinc enzymes, *e.g.* carbonic anhydrase. We developed the tris[imidazolyl-4(5)-yl] phosphane ligands as water-stable and, depending on their substituents, water soluble *N,N,N* ligands (Kunz *et al.* 2003). Their zinc complexes display some esterase-like activity (Kunz& Kläui, 2007). Recently we investigated the coordination behavior of zinc(II) and cobalt(II) complexes of these ligands in the presence of different biologically relevant *N,O* ligands related to the Vahrenkamp-type complexes (Tekeste & Vahrenkamp, 2006). Cobalt(II) resembles in many points the structural coordination chemistry of zinc(II) and often is used to probe the coordination environment in corresponding complexes. UV/Vis data of the title compound indicated the presence of five-and six-coordinate Co(II)-species in solution (Kunz *et al.* 2011).

The molecular structure of the title compound is shown in Figure 1. The coordination polyhedron around the central cobalt(II) atom is about half way from square-pyramidal to trigonal-bipyramidal geometry. The deviation from the tbp geometry is due to the bite of the ligand which allows only for N—M—N angles of up to about 100°. The above mentioned similarity in the structural coordination behavior is shown here, too, as the title compound is isotopic to the corresponding zinc compound (Kunz *et al.*, 2011). In the crystal structure of the title compound the molecules are connected *via* N–H···O hydrogen bonding between the N–H atoms of the imidazolyl substituents in the complex cation and the O-atom of the picolinato ligand of a neighboring complex cation (N2H02···O2', d = 2.786 (3) Å) as well as the O-atoms of nitrate ions (N4H04···O3, d = 2.791 (3) Å, Figure 2). According to these geometric parameters these hydrogen bonds may be classified as medium strong (Steiner, 2002).

### Experimental

The synthesis of the title compound was performed as previously reported (Kunz *et al.* 2011). The title compound was crystallized from methanol solution by slow vapor diffusion of diethyl ether to yield purple crystals.

### Refinement

All CH H atoms were positioned with idealized geometry and refined isotropic with *U*_iso_(H) = 1.2*U*_eq_(C) for C-H and *U*_iso_(H) = 1.5*U*_eq_(C) for CH_3_ groups using a riding model. Atomic coordinates of H atoms of NH groups were refined unrestricted with individual isotropic displacement parameters.

### Crystal data

**Table:**

[Co(C_6_H_4_NO_2_)(C_18_H_27_N_6_P)]NO_3_	*F*(000) = 1252
*M**_r_* = 601.47	*D*_x_ = 1.357 Mg m^−^^3^
Monoclinic, *P*2_1_/*c*	Mo *K*α radiation, λ = 0.71073 Å
*a* = 15.4012 (5) Å	Cell parameters from 6583 reflections
*b* = 10.7035 (3) Å	θ = 3.3–27.2°
*c* = 17.8548 (5) Å	µ = 0.68 mm^−^^1^
β = 90.491 (3)°	*T* = 292 K
*V* = 2943.21 (15) Å^3^	Block, purple
*Z* = 4	0.60 × 0.58 × 0.30 mm

### Data collection

**Table:**

Oxford Diffraction Xcalibur Eos diffractometer	5760 independent reflections
Radiation source: fine-focus sealed tube	4526 reflections with *I* > 2σ(*I*)
Equatorial mounted graphite monochromator	*R*_int_ = 0.022
Detector resolution: 16.2711 pixels mm^-1^	θ_max_ = 26.0°, θ_min_ = 3.3°
ω scans	*h* = −17→18
Absorption correction: gaussian (*CrysAlis PRO*; Oxford Diffraction, 2009)	*k* = −11→13
*T*_min_ = 0.67, *T*_max_ = 0.82	*l* = −22→10
12068 measured reflections	

### Refinement

**Table:**

Refinement on *F*^2^	Primary atom site location: structure-invariant direct methods
Least-squares matrix: full	Secondary atom site location: difference Fourier map
*R*[*F*^2^ > 2σ(*F*^2^)] = 0.042	Hydrogen site location: inferred from neighbouring sites
*wR*(*F*^2^) = 0.086	H atoms treated by a mixture of independent and constrained refinement
*S* = 1.01	*w* = 1/[σ^2^(*F*_o_^2^) + (0.017*P*)^2^ + 2.8*P*] where *P* = (*F*_o_^2^ + 2*F*_c_^2^)/3
5760 reflections	(Δ/σ)_max_ = 0.001
370 parameters	Δρ_max_ = 0.43 e Å^−^^3^
0 restraints	Δρ_min_ = −0.30 e Å^−^^3^

### Special details

**Table:**

Experimental. CrysAlisPro, Oxford Diffraction Ltd., Version 1.171.34.44. Numerical absorption correction based on gaussian integration over a multifaceted crystal model
Geometry. All e.s.d.'s (except the e.s.d. in the dihedral angle between two l.s. planes) are estimated using the full covariance matrix. The cell e.s.d.'s are taken into account individually in the estimation of e.s.d.'s in distances, angles and torsion angles; correlations between e.s.d.'s in cell parameters are only used when they are defined by crystal symmetry. An approximate (isotropic) treatment of cell e.s.d.'s is used for estimating e.s.d.'s involving l.s. planes.
Refinement. Refinement of *F*^2^ against ALL reflections. The weighted *R*-factor *wR* and goodness of fit *S* are based on *F*^2^, conventional *R*-factors *R* are based on *F*, with *F* set to zero for negative *F*^2^. The threshold expression of *F*^2^ > σ(*F*^2^) is used only for calculating *R*-factors(gt) *etc*. and is not relevant to the choice of reflections for refinement. *R*-factors based on *F*^2^ are statistically about twice as large as those based on *F*, and *R*- factors based on ALL data will be even larger.

### Fractional atomic coordinates and isotropic or equivalent isotropic displacement parameters (Å^2^)

**Table:**

	*x*	*y*	*z*	*U*_iso_*/*U*_eq_	
Co1	0.29459 (2)	0.83916 (3)	0.173518 (18)	0.04257 (10)	
P1	0.20777 (5)	0.62495 (7)	0.28876 (4)	0.05045 (18)	
C1	0.32126 (16)	0.6636 (2)	0.30250 (13)	0.0456 (6)	
C2	0.37830 (18)	0.6149 (3)	0.35249 (14)	0.0522 (6)	
H2	0.3672	0.5519	0.3870	0.063*	
C3	0.44493 (17)	0.7603 (2)	0.28778 (14)	0.0475 (6)	
C4	0.21002 (17)	0.5951 (2)	0.18877 (14)	0.0486 (6)	
C5	0.1718 (2)	0.5000 (3)	0.15108 (16)	0.0614 (8)	
H5	0.1336	0.4413	0.1706	0.074*	
C6	0.25358 (18)	0.6041 (2)	0.07286 (14)	0.0499 (6)	
C7	0.16572 (16)	0.7822 (3)	0.29169 (14)	0.0473 (6)	
C8	0.10861 (17)	0.8315 (3)	0.34061 (15)	0.0586 (7)	
H8	0.0803	0.7891	0.3787	0.070*	
C9	0.15266 (17)	0.9811 (3)	0.26536 (14)	0.0523 (7)	
C10	0.51532 (18)	0.8439 (3)	0.26060 (17)	0.0617 (8)	
H10	0.4884	0.9060	0.2275	0.074*	
C11	0.5792 (3)	0.7712 (5)	0.2147 (3)	0.154 (2)	
H11A	0.6242	0.8261	0.1978	0.230*	
H11B	0.5499	0.7352	0.1722	0.230*	
H11C	0.6042	0.7059	0.2448	0.230*	
C12	0.5591 (3)	0.9134 (5)	0.3234 (2)	0.144 (2)	
H12A	0.5165	0.9600	0.3508	0.215*	
H12B	0.6014	0.9698	0.3033	0.215*	
H12C	0.5873	0.8551	0.3564	0.215*	
C13	0.3025 (2)	0.6388 (3)	0.00336 (15)	0.0616 (8)	
H13	0.2719	0.7095	−0.0197	0.074*	
C14	0.3928 (2)	0.6827 (4)	0.02242 (18)	0.0877 (11)	
H14A	0.3897	0.7524	0.0561	0.131*	
H14B	0.4218	0.7078	−0.0225	0.131*	
H14C	0.4246	0.6160	0.0458	0.131*	
C15	0.3043 (3)	0.5351 (4)	−0.05373 (19)	0.1029 (14)	
H15A	0.2460	0.5140	−0.0682	0.154*	
H15B	0.3323	0.4631	−0.0324	0.154*	
H15C	0.3359	0.5622	−0.0969	0.154*	
C16	0.1601 (2)	1.1072 (3)	0.23097 (16)	0.0653 (8)	
H16	0.2069	1.1030	0.1942	0.078*	
C17	0.1845 (3)	1.2070 (4)	0.2869 (2)	0.1028 (13)	
H17A	0.2001	1.2820	0.2608	0.154*	
H17B	0.2329	1.1790	0.3167	0.154*	
H17C	0.1360	1.2235	0.3188	0.154*	
C18	0.0780 (3)	1.1407 (4)	0.1889 (3)	0.153 (2)	
H18A	0.0897	1.2079	0.1548	0.230*	
H18B	0.0344	1.1664	0.2238	0.230*	
H18C	0.0577	1.0693	0.1614	0.230*	
C19	0.35434 (18)	1.0474 (3)	0.08976 (14)	0.0504 (6)	
C20	0.26933 (16)	1.0186 (2)	0.05122 (13)	0.0442 (6)	
C21	0.23571 (19)	1.0905 (3)	−0.00650 (15)	0.0582 (7)	
H21	0.2653	1.1603	−0.0239	0.070*	
C22	0.1571 (2)	1.0558 (3)	−0.03758 (16)	0.0654 (8)	
H22	0.1327	1.1025	−0.0763	0.078*	
C23	0.11511 (19)	0.9523 (3)	−0.01114 (16)	0.0616 (8)	
H23	0.0626	0.9270	−0.0323	0.074*	
C24	0.15199 (17)	0.8861 (3)	0.04739 (16)	0.0562 (7)	
H24	0.1230	0.8166	0.0659	0.067*	
N1	0.36426 (13)	0.75589 (19)	0.26150 (11)	0.0447 (5)	
N2	0.45508 (17)	0.6754 (2)	0.34284 (13)	0.0542 (6)	
H02	0.498 (2)	0.665 (3)	0.3653 (17)	0.072 (11)*	
N3	0.26053 (13)	0.6614 (2)	0.13840 (11)	0.0471 (5)	
N4	0.19990 (18)	0.5066 (2)	0.07917 (14)	0.0629 (7)	
H04	0.180 (2)	0.459 (3)	0.0471 (18)	0.080 (11)*	
N5	0.19332 (13)	0.8781 (2)	0.24447 (11)	0.0466 (5)	
N6	0.10047 (17)	0.9546 (3)	0.32355 (14)	0.0627 (7)	
H06	0.074 (2)	1.005 (3)	0.3463 (17)	0.069 (10)*	
N7	0.22815 (13)	0.91894 (19)	0.07825 (11)	0.0462 (5)	
O1	0.37455 (11)	0.97651 (17)	0.14465 (10)	0.0547 (5)	
O2	0.39853 (14)	1.1354 (2)	0.06762 (11)	0.0740 (6)	
N8	0.0460 (2)	0.3434 (3)	−0.03062 (17)	0.0736 (7)	
O3	0.12255 (18)	0.3765 (3)	−0.03933 (13)	0.0974 (8)	
O4	0.0256 (2)	0.3016 (3)	0.02838 (17)	0.1439 (13)	
O5	−0.00042 (18)	0.3488 (4)	−0.08683 (19)	0.1316 (12)	

### Atomic displacement parameters (Å^2^)

**Table:**

	*U*^11^	*U*^22^	*U*^33^	*U*^12^	*U*^13^	*U*^23^
Co1	0.04483 (19)	0.04102 (19)	0.04190 (19)	−0.00224 (15)	0.00195 (13)	0.00386 (15)
P1	0.0584 (4)	0.0487 (4)	0.0443 (4)	−0.0100 (3)	0.0062 (3)	0.0048 (3)
C1	0.0544 (15)	0.0421 (14)	0.0404 (13)	0.0009 (12)	0.0025 (11)	0.0017 (11)
C2	0.0661 (18)	0.0455 (15)	0.0449 (14)	0.0025 (13)	0.0016 (12)	0.0060 (12)
C3	0.0497 (15)	0.0473 (15)	0.0455 (14)	0.0059 (12)	−0.0001 (11)	0.0019 (12)
C4	0.0579 (16)	0.0415 (14)	0.0466 (14)	−0.0043 (12)	0.0002 (12)	0.0012 (12)
C5	0.077 (2)	0.0508 (17)	0.0564 (17)	−0.0144 (15)	−0.0013 (14)	0.0044 (14)
C6	0.0601 (17)	0.0444 (15)	0.0453 (14)	0.0030 (13)	−0.0022 (12)	0.0005 (12)
C7	0.0413 (14)	0.0550 (16)	0.0456 (14)	−0.0064 (12)	0.0029 (11)	−0.0024 (12)
C8	0.0525 (16)	0.071 (2)	0.0529 (16)	−0.0092 (15)	0.0122 (12)	−0.0059 (15)
C9	0.0507 (16)	0.0590 (18)	0.0472 (15)	0.0075 (13)	−0.0010 (12)	−0.0064 (13)
C10	0.0478 (16)	0.0689 (19)	0.0683 (18)	−0.0029 (15)	−0.0041 (13)	0.0156 (16)
C11	0.116 (4)	0.130 (4)	0.217 (6)	0.008 (3)	0.101 (4)	0.014 (4)
C12	0.167 (5)	0.162 (5)	0.102 (3)	−0.110 (4)	−0.026 (3)	0.020 (3)
C13	0.076 (2)	0.067 (2)	0.0425 (15)	0.0097 (16)	0.0039 (13)	0.0017 (14)
C14	0.095 (3)	0.106 (3)	0.063 (2)	−0.028 (2)	0.0185 (18)	0.003 (2)
C15	0.107 (3)	0.132 (4)	0.070 (2)	−0.022 (3)	0.023 (2)	−0.040 (2)
C16	0.081 (2)	0.0563 (18)	0.0585 (18)	0.0226 (16)	0.0032 (15)	0.0005 (15)
C17	0.145 (4)	0.075 (3)	0.088 (3)	−0.029 (3)	−0.002 (2)	0.002 (2)
C18	0.163 (5)	0.091 (3)	0.204 (6)	0.019 (3)	−0.103 (4)	0.029 (3)
C19	0.0590 (16)	0.0451 (15)	0.0471 (15)	−0.0083 (13)	−0.0044 (12)	0.0039 (12)
C20	0.0513 (15)	0.0415 (14)	0.0399 (13)	0.0001 (12)	−0.0026 (11)	−0.0012 (11)
C21	0.075 (2)	0.0512 (17)	0.0484 (15)	0.0011 (15)	−0.0081 (14)	0.0057 (13)
C22	0.080 (2)	0.064 (2)	0.0523 (17)	0.0168 (17)	−0.0187 (15)	−0.0038 (15)
C23	0.0559 (17)	0.0644 (19)	0.0642 (18)	0.0105 (15)	−0.0167 (14)	−0.0114 (16)
C24	0.0455 (15)	0.0574 (17)	0.0656 (18)	−0.0025 (13)	−0.0040 (13)	−0.0028 (14)
N1	0.0453 (12)	0.0437 (12)	0.0452 (12)	0.0005 (10)	0.0020 (9)	0.0035 (9)
N2	0.0565 (15)	0.0550 (15)	0.0509 (14)	0.0094 (12)	−0.0065 (11)	0.0029 (11)
N3	0.0567 (13)	0.0439 (12)	0.0407 (11)	−0.0043 (10)	0.0020 (9)	0.0015 (10)
N4	0.0872 (19)	0.0533 (15)	0.0480 (14)	−0.0117 (14)	−0.0090 (13)	−0.0062 (12)
N5	0.0450 (12)	0.0492 (13)	0.0454 (12)	0.0043 (10)	0.0003 (9)	0.0000 (10)
N6	0.0578 (15)	0.0702 (19)	0.0603 (16)	0.0101 (14)	0.0102 (12)	−0.0150 (14)
N7	0.0450 (12)	0.0448 (12)	0.0486 (12)	−0.0025 (10)	−0.0024 (9)	−0.0005 (10)
O1	0.0531 (11)	0.0569 (12)	0.0539 (11)	−0.0116 (9)	−0.0122 (8)	0.0139 (9)
O2	0.0810 (14)	0.0689 (14)	0.0719 (13)	−0.0335 (12)	−0.0195 (11)	0.0266 (11)
N8	0.083 (2)	0.0668 (18)	0.0713 (19)	−0.0104 (16)	0.0193 (16)	−0.0039 (15)
O3	0.112 (2)	0.114 (2)	0.0667 (15)	−0.0531 (17)	0.0027 (13)	−0.0108 (14)
O4	0.174 (3)	0.155 (3)	0.104 (2)	−0.012 (2)	0.077 (2)	0.032 (2)
O5	0.0838 (19)	0.191 (4)	0.120 (2)	−0.025 (2)	−0.0192 (18)	0.016 (2)

### Geometric parameters (Å, º)

**Table:**

Co1—O1	1.9885 (17)	C13—C14	1.505 (4)
Co1—N5	2.060 (2)	C13—C15	1.508 (4)
Co1—N3	2.070 (2)	C13—H13	0.9800
Co1—N1	2.094 (2)	C14—H14A	0.9600
Co1—N7	2.154 (2)	C14—H14B	0.9600
P1—C7	1.805 (3)	C14—H14C	0.9600
P1—C1	1.811 (3)	C15—H15A	0.9600
P1—C4	1.814 (3)	C15—H15B	0.9600
C1—C2	1.352 (3)	C15—H15C	0.9600
C1—N1	1.400 (3)	C16—C17	1.508 (4)
C2—N2	1.360 (4)	C16—C18	1.509 (5)
C2—H2	0.9300	C16—H16	0.9800
C3—N1	1.325 (3)	C17—H17A	0.9600
C3—N2	1.347 (3)	C17—H17B	0.9600
C3—C10	1.490 (4)	C17—H17C	0.9600
C4—C5	1.353 (4)	C18—H18A	0.9600
C4—N3	1.389 (3)	C18—H18B	0.9600
C5—N4	1.361 (4)	C18—H18C	0.9600
C5—H5	0.9300	C19—O2	1.229 (3)
C6—N3	1.324 (3)	C19—O1	1.276 (3)
C6—N4	1.337 (4)	C19—C20	1.506 (3)
C6—C13	1.504 (4)	C20—N7	1.334 (3)
C7—C8	1.352 (3)	C20—C21	1.383 (3)
C7—N5	1.397 (3)	C21—C22	1.378 (4)
C8—N6	1.358 (4)	C21—H21	0.9300
C8—H8	0.9300	C22—C23	1.368 (4)
C9—N5	1.323 (3)	C22—H22	0.9300
C9—N6	1.349 (3)	C23—C24	1.381 (4)
C9—C16	1.487 (4)	C23—H23	0.9300
C10—C12	1.501 (5)	C24—N7	1.338 (3)
C10—C11	1.502 (5)	C24—H24	0.9300
C10—H10	0.9800	N2—H02	0.78 (3)
C11—H11A	0.9600	N4—H04	0.82 (3)
C11—H11B	0.9600	N6—H06	0.79 (3)
C11—H11C	0.9600	N8—O4	1.189 (3)
C12—H12A	0.9600	N8—O5	1.229 (4)
C12—H12B	0.9600	N8—O3	1.242 (3)
C12—H12C	0.9600		
			
O1—Co1—N5	118.91 (8)	H14B—C14—H14C	109.5
O1—Co1—N3	139.21 (8)	C13—C15—H15A	109.5
N5—Co1—N3	100.43 (8)	C13—C15—H15B	109.5
O1—Co1—N1	101.22 (7)	H15A—C15—H15B	109.5
N5—Co1—N1	90.61 (8)	C13—C15—H15C	109.5
N3—Co1—N1	87.87 (8)	H15A—C15—H15C	109.5
O1—Co1—N7	78.07 (7)	H15B—C15—H15C	109.5
N5—Co1—N7	92.77 (8)	C9—C16—C17	112.9 (3)
N3—Co1—N7	90.45 (8)	C9—C16—C18	110.8 (3)
N1—Co1—N7	176.45 (8)	C17—C16—C18	111.3 (3)
C7—P1—C1	97.42 (12)	C9—C16—H16	107.2
C7—P1—C4	101.68 (12)	C17—C16—H16	107.2
C1—P1—C4	98.45 (12)	C18—C16—H16	107.2
C2—C1—N1	108.1 (2)	C16—C17—H17A	109.5
C2—C1—P1	128.5 (2)	C16—C17—H17B	109.5
N1—C1—P1	123.43 (18)	H17A—C17—H17B	109.5
C1—C2—N2	107.1 (2)	C16—C17—H17C	109.5
C1—C2—H2	126.5	H17A—C17—H17C	109.5
N2—C2—H2	126.5	H17B—C17—H17C	109.5
N1—C3—N2	109.7 (2)	C16—C18—H18A	109.5
N1—C3—C10	126.1 (2)	C16—C18—H18B	109.5
N2—C3—C10	124.2 (2)	H18A—C18—H18B	109.5
C5—C4—N3	107.8 (2)	C16—C18—H18C	109.5
C5—C4—P1	127.6 (2)	H18A—C18—H18C	109.5
N3—C4—P1	124.27 (19)	H18B—C18—H18C	109.5
C4—C5—N4	106.8 (3)	O2—C19—O1	124.8 (2)
C4—C5—H5	126.6	O2—C19—C20	119.4 (2)
N4—C5—H5	126.6	O1—C19—C20	115.8 (2)
N3—C6—N4	109.4 (2)	N7—C20—C21	122.6 (2)
N3—C6—C13	125.3 (2)	N7—C20—C19	114.4 (2)
N4—C6—C13	125.2 (2)	C21—C20—C19	123.0 (2)
C8—C7—N5	107.8 (2)	C22—C21—C20	118.2 (3)
C8—C7—P1	128.2 (2)	C22—C21—H21	120.9
N5—C7—P1	123.83 (18)	C20—C21—H21	120.9
C7—C8—N6	107.0 (3)	C23—C22—C21	119.7 (3)
C7—C8—H8	126.5	C23—C22—H22	120.2
N6—C8—H8	126.5	C21—C22—H22	120.2
N5—C9—N6	109.2 (3)	C22—C23—C24	118.9 (3)
N5—C9—C16	127.0 (2)	C22—C23—H23	120.5
N6—C9—C16	123.8 (3)	C24—C23—H23	120.5
C3—C10—C12	112.2 (3)	N7—C24—C23	122.0 (3)
C3—C10—C11	110.4 (3)	N7—C24—H24	119.0
C12—C10—C11	111.9 (4)	C23—C24—H24	119.0
C3—C10—H10	107.4	C3—N1—C1	106.6 (2)
C12—C10—H10	107.4	C3—N1—Co1	136.34 (17)
C11—C10—H10	107.4	C1—N1—Co1	116.81 (16)
C10—C11—H11A	109.5	C3—N2—C2	108.5 (2)
C10—C11—H11B	109.5	C3—N2—H02	125 (2)
H11A—C11—H11B	109.5	C2—N2—H02	127 (2)
C10—C11—H11C	109.5	C6—N3—C4	107.1 (2)
H11A—C11—H11C	109.5	C6—N3—Co1	135.34 (18)
H11B—C11—H11C	109.5	C4—N3—Co1	114.51 (16)
C10—C12—H12A	109.5	C6—N4—C5	108.8 (2)
C10—C12—H12B	109.5	C6—N4—H04	131 (2)
H12A—C12—H12B	109.5	C5—N4—H04	120 (2)
C10—C12—H12C	109.5	C9—N5—C7	107.1 (2)
H12A—C12—H12C	109.5	C9—N5—Co1	134.80 (18)
H12B—C12—H12C	109.5	C7—N5—Co1	117.28 (16)
C6—C13—C14	111.1 (2)	C9—N6—C8	108.8 (3)
C6—C13—C15	112.9 (3)	C9—N6—H06	124 (2)
C14—C13—C15	111.1 (3)	C8—N6—H06	127 (2)
C6—C13—H13	107.2	C20—N7—C24	118.6 (2)
C14—C13—H13	107.2	C20—N7—Co1	112.25 (16)
C15—C13—H13	107.2	C24—N7—Co1	129.02 (18)
C13—C14—H14A	109.5	C19—O1—Co1	119.43 (16)
C13—C14—H14B	109.5	O4—N8—O5	125.8 (4)
H14A—C14—H14B	109.5	O4—N8—O3	118.5 (4)
C13—C14—H14C	109.5	O5—N8—O3	115.5 (3)
H14A—C14—H14C	109.5		

### Hydrogen-bond geometry (Å, º)

**Table:**

*D*—H···*A*	*D*—H	H···*A*	*D*···*A*	*D*—H···*A*
N2—H02···O2^i^	0.78 (3)	2.01 (3)	2.786 (3)	174 (3)
N4—H04···O3	0.82 (3)	1.98 (3)	2.791 (3)	169 (3)

Symmetry code: (i) −*x*+1, *y*−1/2, −*z*+1/2.
